# Pituitary adenoma with cavernous sinus compartment penetration and intracranial extension: surgical anatomy, approach, and outcomes

**DOI:** 10.3389/fonc.2023.1169224

**Published:** 2023-05-18

**Authors:** FuMing Yang, YunKe Bi, QiangYi Zhou, HongChan Li, YaJun Xue, QingWei Zhu, Jian Yin, ZhiYu Wang, MeiQing Lou

**Affiliations:** ^1^ Department of Neurosurgery, Shanghai General Hospital, Shanghai JiaoTong University School of Medicine, Shanghai, China; ^2^ Department of Radiology, Shanghai General Hospital, Shanghai JiaoTong University School of Medicine, Shanghai, China

**Keywords:** intracranial extension, cavernous sinus compartments penetration, pituitary adenoma growth corridor, endonasal endoscopic surgery, outcomes

## Abstract

**Objectives:**

To understand the different characteristics and growth corridors of knosp grade 4 pituitary adenomas (Knosp4PA) with cavernous sinus (CS) compartments penetration and intracranial extension, aiming to improve the safety, effectiveness, and total resection rate of surgery.

**Methods:**

A case series of 120 Knosp4PA patients with 187 invaded compartments were retrospectively reviewed. A novel surgery-relevant grading system was proposed according to the CS penetrating features. The details of approach drafting, risk prediction, and complication avoidance were analyzed and integrated through illustrated cases.

**Results:**

All enrolled tumor was Knosp4PA which was derived from Knosp subgrades 3A(62.5%) and 3B(37.5%). Based on the tumor growth pathway and its relevant features, five subclassifications of intracranial extension(n=98,81.7%) were classified, which derived from the superior (Dolenc’s and Oculomotor subtype, 5% and 24.2%), lateral (Parkinson’s subtype,18.3%), and posterior (cerebral peduncle and Dorello’s subtype, 5.8% and 1.7%) CS compartment penetration. The size of intracranial extension is assessed by Lou’s scale proposed here based on preoperative MRI characteristics. Under Lou’s scale, the gross total rate (GTR) decreased (82%, 53%, 22%, and 19%) with grades increased (grade 0,1,2,3, respectively), and presents significant difference between the four groups (p=0.000), as well as between single and multiple compartments involved (p=0.001). Preoperative cranial nerve deficits included the optic nerve (53%), oculomotor nerve (24.2%), and abducent nerve (4.2%), with an overall rate of visual function improvement in 68.1%. Postoperative complications of transient diabetes insipidus, cerebrospinal fluid (CSF) leakage, and cranial nerve deficits were 6.7%, 0.8%, and 0%. No new cranial nerve deficits occurred. The mortality rate was 0.8%.

**Conclusion:**

The concept of “penetration” refines the extracavernous growth pattern, and the five intracranial subclassifications help to understand the potential extension corridors, enhancing adequate exposure and targeted resection of Knosp4PA. This grading system may benefit from its predictive and prognostic value, from which a higher GTR rate can be achieved.

## 1 Introduction

Despite advances in the endoscopic endonasal approach (EEA), complex pituitary adenomas invading and penetrating beyond the cavernous sinus remain challenging in neurosurgery (1–3). Tumors may fill the CS cavity and extend outside through a slit-like corridor of the cranial nerves (4–7), leading to the internal carotid artery (ICA) and cranial nerve displacement and leaving residual tumor if the unitary EEA is taken. At that point, a “salvage” transcranial route is necessary for reexploration whenever complications occur. Studies of EEA have recently reported successful resection of tumors extending into the oculomotor triangle (8) and posterior triangle (9), which offer choices for experienced centers. Nevertheless, the following problems can be faced in clinical practice: 1) From which part should the resection be initiated? An unfamiliarity with Penetration may lead to inadvertent manipulation. 2) Which strategy should be chosen? Low adequate exposure and purposeful resection may leave residual tumor, which results in recurrence and imposes a financial and psychological burden on patients. In this study, we proposed a subclassification of Knosp4 pituitary adenomas that have penetrated the intradural space. We also introduced EEA strategies that took advantage of the natural growth corridor to improve the resection rate. The clinical features, surgical strategy, and outcomes were analyzed and compared between subgroups, and technical nuances are illustrated in five cases.

## 2 Materials and methods

### 2.1 Patient population

A retrospective database study was conducted from January 2017 to December 2020. In this all-Knosp grade-4 pituitary adenoma series, a total of 120 patients with 187 sites of CS compartment invasion were collected and selected, all CS penetration and intracranial extension were confirmed by EEA or microscopic-TC. The cases that met the following inclusion criteria were analyzed:

1. CS invasiveness and Penetration were assessed by a team of 1 neuroradiologist + 1 neurosurgeon based on MRI, and intraoperative findings confirmed Penetration.
2. The patient underwent pre- and postoperative endocrine assessments (PRL, GH, IGF, ACTH, cortisol, TSH/T3/T4, FSH/LH levels) and ophthalmologic examinations (visual acuity testing, eye movements, perimetry for visual field assessment, and fundoscopy).
3. All procedures were performed by the same surgeon (MeiQing Lou).
4. A CT scan assessed sinus pneumatization.

Informed consent was obtained from all patients. Per the principles of the Declaration of Helsinki, our ethical review committee approved the study.

### 2.2 Criteria for surgery

(1) Knosp4PA appeared to invade the cavernous space, penetrated, and caused compression symptoms; (2) the functional pituitary tumor resulted in hormonal imbalance that could not be controlled by pharmacotherapy, i.e., Parlodel (Novartis Farma S.P.a.) for at least a 3-month course in patients with prolactinoma; Dostinex was not available in China.

### 2.3 Growth pattern of intracranial extension

**Figure 1:**
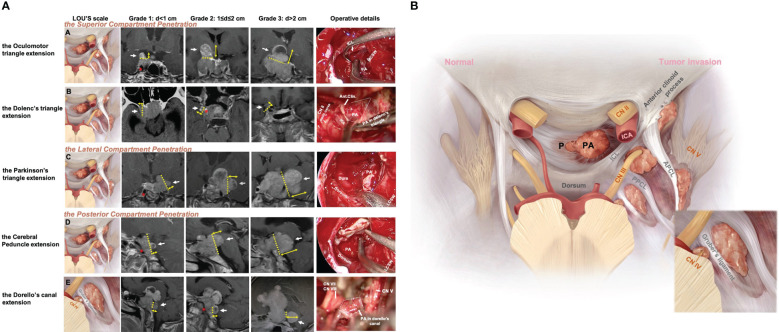
Five patterns of Knosp4PA with cavernous sinus compartment penetration and intracranial extension. **(A)**, Left column, Graphic schemes, the white star indicates penetration region, i.e., extension into (row-A) the oculomotor triangle, (row-B) the Dolenc’s triangle, (row-C) the Parkinson’s triangle, (row-D) the cerebral peduncle, (row-E) and the Dorello’s canal.; Middle column, sellar region MRI images red star indicates encased ICA; Right column, endoscopic and transcranial views. A dashed line indicates penetration entrance. **(B)**, Graphic schemes show the CS penetration pattern (right) compared to the normal anatomical structure of the pituitary(left) located in the sella region. The lower right figure shows a zoomed view of Dorello’s canal penetration. The EXTENSION GRADE was estimated practically by preoperational MRI based on the maximum diameter from the growth entrance, which was delimited superiorly (APCL-PPCL-ICL), laterally (CNIV (trochlear nerve)-CNV1(optical nerve)-PPCL), and posteriorly (PPCL-dorsum-Gruber’s ligament) and was categorized as grade 0 (CS invasion without Penetration), grade 1 (Penetration <1 cm), grade 2 (Penetration =1–2 cm), or grade 3 (penetration >2 cm). APCL, anterior petroclinoidal ligament; CN, cranial nerve; CS, cavernous sinus; ICA, internal carotid artery; ICL, interclinoidal dural ligament; LCSW, lateral CS wall; MCSW, medial CS wall; P, normal pituitary; PA, pituitary adenoma; PPCL, posterior petroclinoidal ligament.

The 120 (187 sites) Knosp4PAs were subclassified into superior, lateral, and posterior CS penetration based on the preoperative MRI characteristics, of which 5 CS-related intracranial extension regions were specified from intraoperative findings (**Figures 1A, B**).

#### 2.3.1 The superior penetration

**Figure 2:**
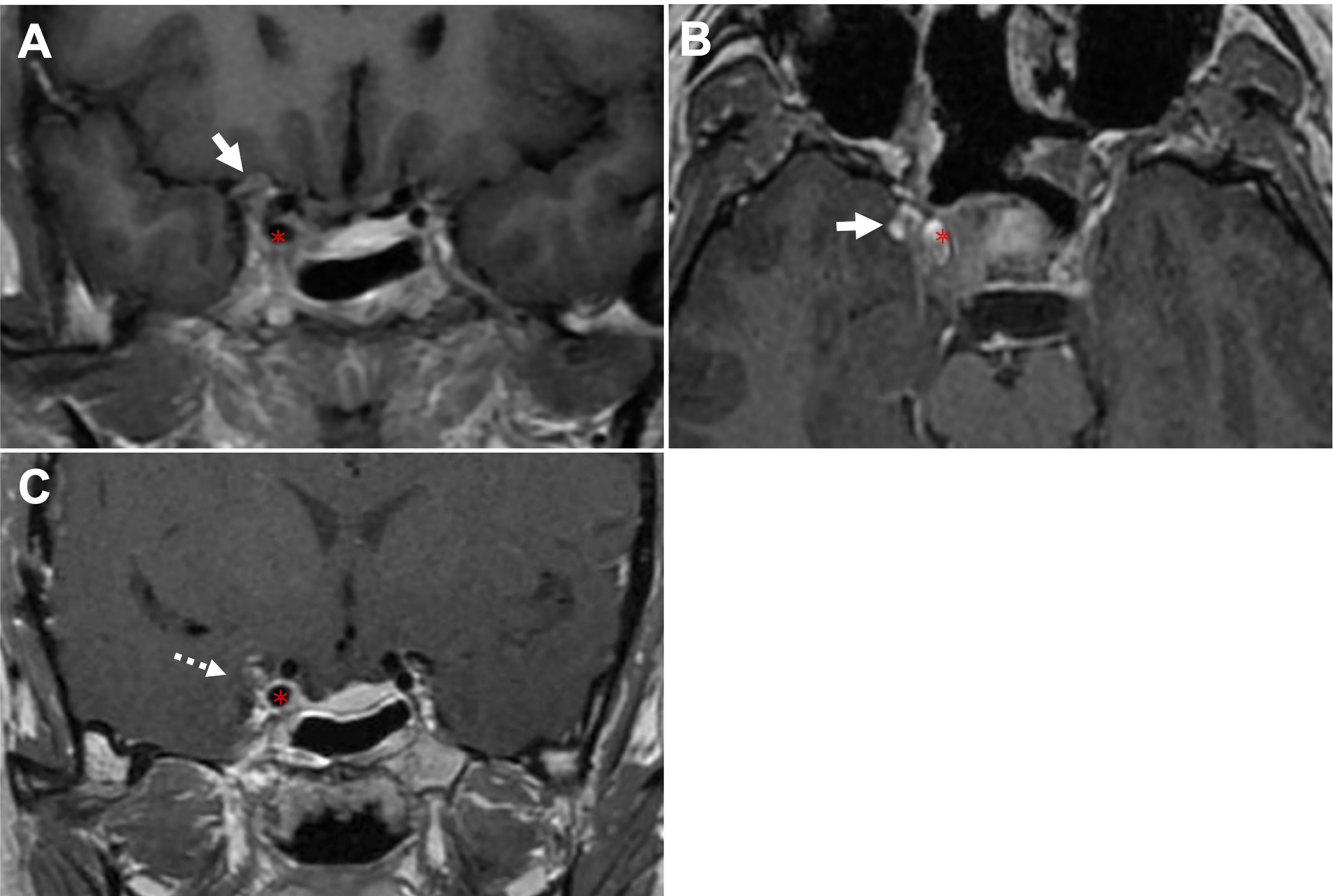
CASE1, The Dolenc’s triangle penetration. A 44-year-old woman presented with a pituitary adenoma. **(A, B)**, Preoperative coronal **(A)** and axial **(B)** T1-weighted contrast-enhanced MRI showing a pituitary adenoma with right-side cavernous sinus invasion (Knosp grade 4) and extension into the Dolenc’s triangle. The tumor is located superior (arrow, in **A**) and lateral (arrow, in **B**) to ICA (asterisk). **(C)**, Postoperative coronal T1-weighted contrast-enhanced MRI showing NTR of the tumor following TC, removal of the anterior clinoid process can expand anterior skull base surgical corridors, we achieved near-total resection with residual tumor (dotted arrow) wrapped ICA. ICA, internal carotid artery; TC, transcranial approach.

The Dolenc’s triangle and the Oculomotor triangle extension originating from the superior compartment penetrate. The upper compartment is above the horizontal cavernous ICA and posterior to the anterior genu. The PA was identified as penetrating the oculomotor triangle (roof of the CS) and extending toward the basal ganglia (cases 4, 5) beyond the anatomic limits by the anterior petroclinoid ligament (APCL) laterally and the interclinoid ligament (ICL) medially. It traveled within this channel and was quickly displaced by tumor compression (cases 2, 3, 5). Further tumor invasion could involve the anterior choroidal artery. It could invade the cortex of the uncus-parahippocampal gyrus (case 3). Extension into Dolenc’s triangle could be identified when the tumor entered the posterior-superior part of the clinoid space, causing bone destruction of the anterior clinoid process (case1, **Figure 2**).

#### 2.3.2 The lateral penetration

The Parkinson’s triangle extension originating from the lateral compartment penetrates. The lateral compartment is located lateral to the cavernous ICA and is bounded by the lateral wall of the CS. The identified PA penetration is associated with Parkinson’s triangle, causing trochlear and ophthalmic nerve displacement. Further tumor invasion can medially compress the temporal lobe and temporal pole (case 4). This region is delineated superiorly by the APCL and inferiorly by the petrolingual ligament and communicates with the inferior compartment as a potential pathway for tumor invasion.

#### 2.3.3 The posterior penetration

The cerebral peduncle and Dorello’s canal extension originating from the posterior compartment penetrate. The posterior compartment is located behind the short vertical segment and posterior genu of the cavernous ICA. The petrosphenoidal (Gruber’s) ligament lies above Dorello’s canal (**Figure 1A**, row E; case 5) and abducens nerve (CN VI) and is inferior to the posterior wall from the dorsal sellar region to the petrous apex. Opening the base of Meckel’s cavity can expose the growth corridor delimited by Gruber’s ligament, PPsb, petroclival fissure, and petrous apex. Tumor extension above Gruber’s ligament results in compression of the ventral aspect of the cerebral peduncle (**Figure 1A**, row D; case 3).

#### 2.3.4 The intracranial extension grade

A newly proposed Lou’s grading system was used for intracranial extension size evaluation. This parameter estimated the maximum diameter(d) of the intracranial perforation portion that exceeds the CS dura margins, grade1 means d<1cm, grade2 means 1≤d ≤ 2cm, grade3 means d>2cm. A special type “grade 0” was used to describe the behavior of CS invasion when PA was limited by the CS margin and absent of perforation. The well described CS margin was categorized by referencing to the corridor of penetrate as the superior (APCL-PPCL-ICL), lateral (CNIV (trochlear nerve)-CNV1(optical nerve)-PPCL), and posterior (PPCL-dorsum-Gruber’s ligament) (**Figure 1**).

### 2.4 Operative details of intracranial extension

#### 2.4.1 Surgical approach selection of intracranial extension

The principle is making individualized operative choices from the findings on the growth patterns of intracranial extensions, their relationship to the ICA, the ligamentous connections inside the CS, and the extension grade. The standard endonasal endoscopic surgical approach to the sphenoid sinus is performed as described (10). Once the sphenoid sinus opening has been maximized, extensive sellar and parasellar exposure, including the anterior wall of the CS and paraclinoid segment of the ICA, is completed.

To detect the superior penetration and extension, the bone covering the paraclinoidal ICA and anterior wall of the CS is removed. This allows medial-to-lateral displacement of the ICA for direct surgical dissection within this region. The transcavernous approach (5) as Fernandez-Miranda’s fashion was performed for the medial wall of the CS(MCSW) mobilization and ICA dissection. Through the medial wall of CS, the superior compartment can be accessed uptowards. Tumors in this region often find a most vulnerable portion just lateral to the anterior genu of the ICA to cause penetration. Once the paraclinoidal ICA, and interclinoidal ligament (ICL, the key landmark of roof) are identified, the dura of the oculomotor triangle can be opened for safe and complete resection. Transection of the ICL may sometimes be needed to provide a widen vision for tumor portions that not easily accessible.

To seek the lateral penetration and extension, a surgical corridor needs to be developed between the anterior genu/horizontal ICA and the CNs in the lateral wall of the CS(LCSW). Tumors between cavernous ICA and CNs (oculomotor, trochlear and abducens) can be carefully removed with the assistance of electrostimulation. Note that the abducens nerve may be embedded within the tumor. For perforations exceed the lateral compartment, where the LCSW is commonly involved, so the STR is determined to avoid CNs/ICA injury. For large extensions (≥grade 3), the planned-stage transcranial surgery by Dolenc approach was used to remove the intracranial residual tumor remained in superolateral clinoid space (the dolenc’s triangle) and in the Parkinson’s triangle.

To trace the posterior penetration and extension, extensive bone removal is required to uncover the anterior wall of the CS and to expose the entrance of ICA into the CS from the medial space. After this work, surgical access posterior to the cavernous ICA can be gained by lateral mobilization of the short vertical and posterior genu of ICA subsegment. Though this route, the tumor within posterior CS compartment can be easily removed. After transposition of the inner portion, the fenestrated dura matter serves as a reference for the location of tumor penetration site. The posterior extensions basically progress in a posterior direction, all the way to the posterior clinoid process and dorsum sellae. Following this path, tumor compressed the cerebral peduncle can be found. And/or after opening the Meckel’s cavity, the gruber’s ligament was the first to be encountered, but surgery to expose this part is often difficult. Following transection of this ligament, tumor toward the dorello’s canal can be dissected. For large extensions (≥grade 3), the planned-stage transcranial surgery by Kawase or pre-sigmoid approach was used to remove the intracranial residual tumor remained.

#### 2.4.2 Prevention of surgical-related complications

Neuronavigation and intraoperative electroencephalography monitoring were routinely used to maximize safety. Nasoseptal flap preparation and sphenoidotomy were performed as is standard (10). To prevent post-operational nose bridge collapse, we preserved the cut edge by 1.5 cm and above to the nasal bone. Careful attention should be paid to the identification and protection of ICA protrusions, the optic canal protrusions and the medial and lateral optic nerve-internal carotid recess (MOCR/LOCR). Adhesions between the tumor and its capsule should be separated with blunt dissection and preserve the utmost integrity of the membrane structure as much as possible. This carefully manipulation is useful for postoperative pituitary function maintenance, as well as reduce the incidence of intracranial infection and cerebrospinal fluid leakage. Reconstruction of skull base was performed with standard 3F technique (11) when CSF leakage is suspected. During the procedure, we emphasized the sufficient reduction of trabeculae structures on the sellar floor, this allows a closely overlap of fascia and pedicle flap. Dissection along the tumor pseudocapsule is necessary to reduce possible incidence of residue. Any tumors’ tight adhesion with vascular and neural structures should be sharply dissected. If a high risk of intraoperative ICA injury was anticipated, the subtotal resection (STR) is acceptable in these regions, and residual tumor is recommended to be managed by further stereotactic radiotherapy. When manipulate the extension regions, identification and protection of the cranial nerve (oculomotor, abducens, and optic nerve), basilar artery and branches, and hypothalamus should be carefully concerned.

### 2.5 Neuroradiological assessment

The degree of surgical resection was assessed by postoperative MRI: gross total resection (GTR) meant no residual tumor; subtotal resection (STR) meant tumor elimination ≥ 80%; partial resection meant tumor elimination <80%.

### 2.6 Follow-up

Postoperative MRI was done three months postoperatively to search for residual tumor. Nasal endoscopy was performed in the first month postoperatively to assess nasal complications such as bleeding, crusting, and development of atrophic rhinitis. The hormone examination was repeated two days and two weeks postoperatively. An ophthalmic evaluation was performed in the immediate postoperative period and three months postoperatively. Patients were assessed for cerebrospinal fluid leakage and intracranial infection at every visit. Stereotactic radiotherapy is recommended if the residual tumor enlarged during any follow-up time.

### 2.7 Statistical analysis

Statistical analysis of categorical variables between the groups was performed using the chi-square test (χ²). A two-tailed P value <0.05 was considered significant. Data were analyzed using SPSS version 25.0 (IBM Corporation).

## 3 Results

### 3.1 Baseline characteristics

Patient and tumor characteristics are shown in **Tables 1**, **2**. The patients included 71 (59%) males and 49 (41%) females (1.45:1); the mean age was 32.6 ± 17.2 (24-67) years. The tumor diameter was 37.0 ± 13.8 (15-90), and the tumors were macroadenoma (n=72) and giant adenoma (n=48). Hormonal subtypes were non-functional (53, 44.2%), PRL (20, 16.6%), GH (14, 11.7%), FSH (11, 9.2%), ACTH (3, 2.5%), TSH (3, 2.5%) and multifunctional adenoma (13, 10.8%). The mean follow-up time was 48.5 ± 25.3 (7-95) months. The most common presenting symptoms were headache (n=96, 80%), followed by decreased visual acuity (n=73, 61%), visual field deficits (n=63, 53%), endocrinopathy (n=54, 45%), nasal obstruction (n= 42, 35%). Preoperative Penetration related cranial nerve deficits were in the optic nerve (n=63, 53%), oculomotor nerve (n=29, 24.2%), and abducent nerve (n=5, 4.2%).

**Table 1:** Patient and tumor characteristics.

Characteristic	Value
Sex (male, female. [ratio])	71, 49 (1.45:1)
Age (median ± SD [range]) (yrs)	32.6 ± 17.2(24-67)
Size (no.)	
microadenoma (<10mm)	0
macroadenoma (10-40mm)	72
giant (>40mm)	48
diameter (median ± SD [range]) (mm)	37.0 ± 13.8(15-90)
Hormonal subtype (no. [%])	
non-functional	53(44.2)
PRL cell adenoma	20(16.6)
GH cell adenoma	14(11.7)
FSH cell adenoma	11(9.2)
ACTH cell adenoma	3(2.5)
TSH cell adenoma	3(2.5)
multifunctional adenoma	13(10.8)
mixed with blood/apoplexy	3(2.5)
Follow-up time (median ± SD [range]) (mon)	48.5 ± 25.3(7-95)

**Table 2:** Clinical presentation.

clinical features	Grade 0 (22)	Grade 1 (55)	Grade 2 (27)	Grade 3 (16)
decreased visual acuity	20	27	15	11
visual field deficits	7	21	21	14
other cranial nerve deficits				
third nerve	–	14	11	4
sixth nerve	–	1	2	2
endocrinopathy	3	30	15	6
headaches	17	49	17	13
nasal obstruction	5	14	17	6

### 3.2 FIVE CS-penetration-related intracranial extension regions

Tumor growth characteristics are shown in **Table 3**. In this series, by Lou’s scale, 22 patients were grade 0, which defined as CS invasion and absent of penetration, and 55, 27, and 16 patients were in grades 1, 2, and 3, respectively. Take all grades in count, the most commonly involved compartment was the superior (82 by sites, 68.3%), followed by the lateral (71 by sites, 59.2%) and posterior (34 by sites, 28.3%), of which 67 patients (55.8%) were single compartment involved, and 53 patients (44.2%) were multiple (two or three). After tumor penetrate the CS compartments’ dura, five intracranial extension types were identified, included two types originating from the CS superior compartment: the oculomotor (n=29, 24.2%) and the Dolenc’s (n=6, 5%); one types originating from the CS lateral compartment: the Parkinson’s (n=22, 18.3%); and two types originating from the CS posterior compartment: the cerebral peduncle (n=7, 5.8%) and the Dorello’s canal (n=2, 1.7%).

**Table 3:** Growth pattern on magnetic resonance imaging by Knosp’s and Lou’s and grading system.

Characteristic	Number (percentage%)
Parasellar invasion (Knosp’s)	
Grade 1-3	0
Grade 4	120(100)
Relationship with ICA/Knosp’s subgrade	
Grade 3A	75(62.5)
Grade 3B	45(37.5)
CS compartment invasion and absent of penetration (grade 0, Lou’s)	22(18.3) patients with 33 CS compartments/sites
superior compartment	17(14.2) sites
lateral compartment	11(9.2) sites
posterior compartment	5(4.2) sites
CS compartment penetrating and intracranial extensions (Lou’s)	98 (81.7) patients with 154 CS compartments/sites
the Superior penetration	65(54.2) sites
**Grade 1**	43(35.8)
**Grade 2**	15(12.5)
**Grade 3**	7(5.8)
Intrcranial extension regions	
*(type 1) the oculomotor triangle extension (basal ganglion directivity)*	29(24.2)
*(type 2) the Dolenc’s triangle extension*	6(5)
the Lateral penetration	60 (50) sites
Grade 1	33(27.5)
Grade 2	19(15.8)
Grade 3	8(6.7)
Intrcranial extension regions	
*(type 3) the Parkinson’s triangle extension*	22(18.3)
the Posterior penetration	29(24.2) sites
Grade 1	16(18.3)
Grade 2	8(6.7)
Grade 3	3(2.5)
Intrcranial extension regions	
*(type 4) the cerebral peduncle compression*	7(5.8)
*(type 5) the Dorello’s canal extension*	2(1.7)

### 3.3 Surgical results of intracranial extension

The extent of tumor removal is summarized in **Tables 4**, **5**. In this series, GTR decreased (82, 53, 22, and 19%) with increasing scores of GRADE (grade0,1,2,3), and the statistical difference was shown in GTR between grade0 and grade2 (p<0.05), and between grade0 and grade3 (p<0.05); In addition, the involvement of single and multiple CS compartment also affects GTR (p=0.001). It suggests that GTR is significantly more difficult to achieve with multiple CS compartment invasions and larger tumor penetration grade (>2 cm, grade 2).

**Table 4:** Extent of Excision.

Grade	CS invasion compartment	Number of patients (120 in total)		
		GTR	NTR	STR
**0**	single	13	1	–
	multiple	5	3	–
1	single	25	12	–
	multiple	4	9	5
2	single	4	3	2
	multiple	2	7	9
3	single	1	4	2
	multiple	2	3	4
**total (%)**	single	44(36.7)	19(15.8)	4(3.3)
	multiple	16(13.3)	19(15.8)	18(15)

**Table 5:** Surgical approach.

Surgical Procedure	Number of patients (120 in total)			
	Grade 0 (22)	Grade 1 (55)	Grade 2 (27)	Grade 3 (16)
EEA	22	47	25	15
EEA+TC	–	3	2	1
TC	–	5	–	–
**Surgery Time(primary)**	22	55	24	13

A total of 109 (90%) patients received one-time surgery; 11 (10%) received staged surgery. According to our strategy, EEA was adopted in grade 0(100%), 86% in grade 1, 93% in grade 2, and 94% in grade 3. While EEA combined staged TC was performed in grade 0(0%), grade 1 (5.5%), grade 2 (7.4%), and grade 3 (6.25%). Pure TC was done in 5 cases, where most cases were Dolenc’s triangle extension.

### 3.4 Outcome, complications, and follow-up

The surgical results and complications are summarized in **Table 6**. Visual improvement in 49 cases (68.1%). Postoperative complications included transient diabetes insipidus in 8 patients (6.7%) and rhinorrhea in 1 patient (0.8%) 3 days postoperatively who had CSF leakage. Surgery for the repair of a CSF leak was done after a delay. One (0.8%) patient had cerebral infarction. No new cranial nerve deficits developed. Of the 22 patients with incomplete tumor resection, five patients with functional PAs received medical treatment, and 17 (2 functional PA and 15 nonfunctional-PA) received radiotherapy. The mortality was 1.96% (1 patient).

**Table 6:** Postoperative outcomes and complications.

clinical features	value (no./total [%])
visual improvement	49/72(68.1)
postoperative complications	10/120(8.3)
CSF leakage	1(0.8)
cerebral infarction	1(0.8)
new cranial nerve deficits	0
diabetes insipidus	8(6.7)
transient	8
permanent	0
mortality	1(0.83)

### 3.5 Knosp4PA with cavernous sinus penetration and intracranial extension: case series

**Figure 3:**
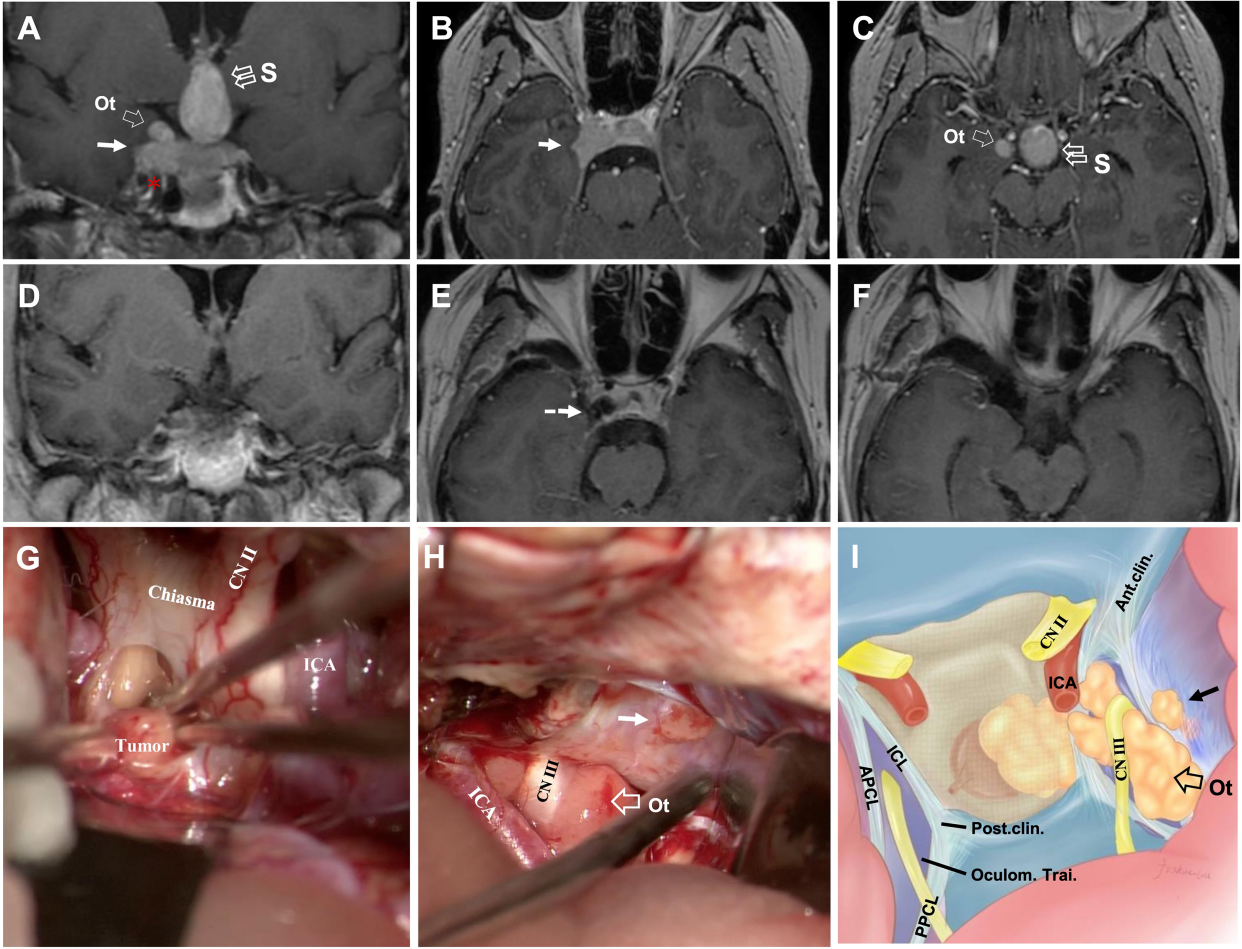
CASE2, The superior (midline+oculomotor triangle)-lateral compartment penetration. A 43-year-old woman with a giant non-functional pituitary adenoma. CC: acute-onset headache and visual field defects (bitemporal hemianopia). **(A–C),** Preoperative coronal **(A)** and axial **(B, C)** T1-weighted contrast-enhanced MRI showing primary tumor with right-side cavernous sinus invasion (Knosp grade 4) and extension through the superior with a midline (“S,” double open arrow) and oculomotor triangle (“Ot,” single open arrow), in **(A, C)** and lateral (solid arrow, in **A, B**) penetration, which present the exact relationship of ICA (asterisk, in **A**). **(D–F),** Postoperative T1-weighted contrast-enhanced MR images showing total resection of the tumor (dotted arrow, in **E**). **(G–I)** Intraoperative findings. Presence of relationship between tumor and optic chiasm, optic nerve (CN II), and ICA **(G)**. The oculomotor nerve (CN III) is displaced superiorly and in direct connection with the superior aspect of the tumor, and the presence of a tumor with an oculomotor triangle and lateral (solid arrow) CS extension **(H)**. Artistic illustration of the typical pattern of an oculomotor triangle (open arrow) and lateral (solid arrow) extension of adenomas outside the cavernous sinus. Vertical view simulating an trancranial approach using a straight endoscope **(I)**. ICA, internal carotid artery; Ot, oculomotor triangle.

**Figure 4:**
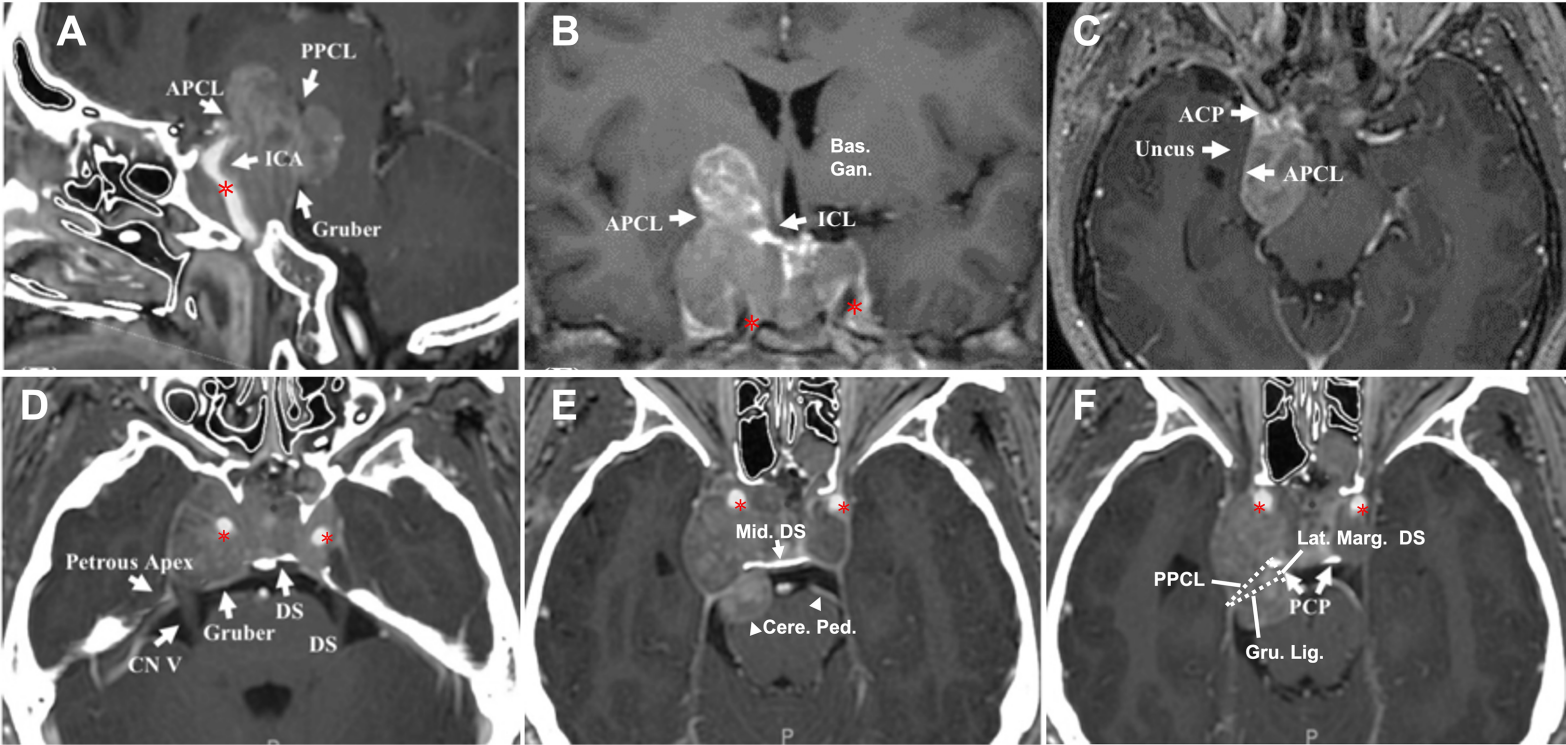
CASE3, The superior-posterior compartment extension. A 66-year-old woman with a giant pituitary adenoma. **(A–C),** Preoperative sagittal **(A)**, coronal **(B)**, and axial **(C)** T1-weighted contrast-enhanced MRI showing a pituitary macroadenoma with bilateral cavernous sinus invasion (Knosp grade 4) and extension through the superior, formed a “cauliflower-like” shape **(A)** and posterior **(B)** but not lateral **(C)** compartment. Note tumor expanded excess of the APCL **(A, B)** and ICL **(B)** superiorly and excess of the PPCL posteriorly **(A)** but was limited by APCL laterally **(C)**. The superior aspect of the tumor compressed basal ganglia **(B)**; the posterior-inferior aspect caused compression of the parapeduncular space **(C)**. **(D–F),** Preoperative axial T1-weighted contrast-enhanced MRI at the dorsum sella root section **(D)**; 5 mm above D at the middle dorsum sellar section **(E)**; and 6 mm above **(D)** at the PCP section **(F)**. **(D)** Intact dorsum sella, the petrous apex was seen, Gruber’s ligament is attached to the lateral dorsum sella, without displacement of bilateral CN V. **(E)** Posterior aspect of tumor excess posterior compartment dorsally of the Gruber’s ligament and compressed the ventral aspect of the cerebral peduncle (arrowhead) directly. **(F)** The typical pattern of posterior triangle invasion, which is excessing the triangle composed of PPCL superolateral, Gruber’s ligament superior, and the lateral margin of the dorsum sellae medially. Note the intimate relationship between ICA (asterisk) and the anteromedial aspect of the tumor. ACP, anterior clinoid process; APCL, anterior petroclinoidal ligament; DS, dorsum sellae; ICA, internal carotid artery; ICL, interclinoidal dural ligament; PCP, posterior clinoid process; PPCL, posterior petroclinoidal ligament; bas. gang., basal ganglia; cere. ped., cerebral peduncle; Gru. Lig., Gruber’s ligament, petrosphenoidal ligament; lat. marg., lateral margin; mid., middle.

**Figure 5:**
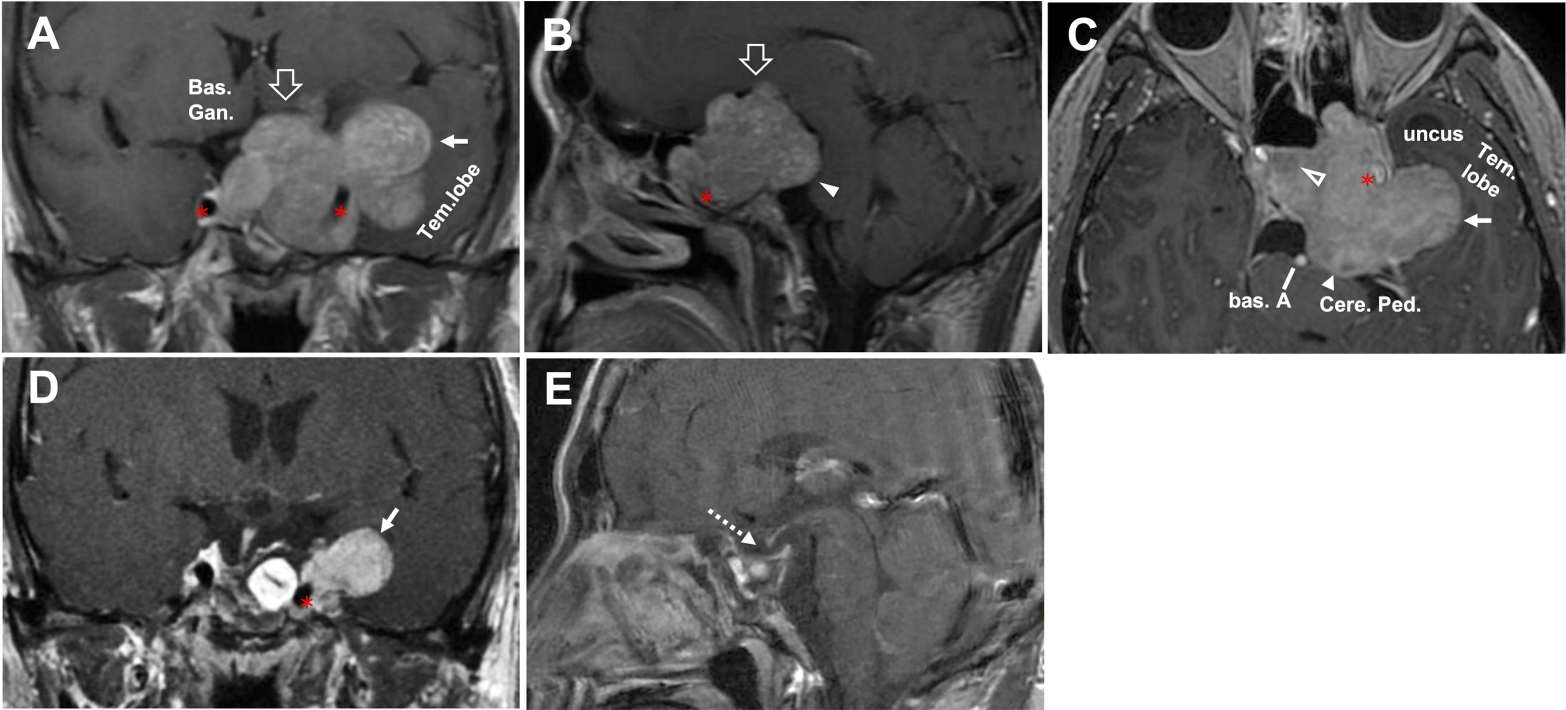
CASE4, The superior-posterior-lateral compartment extension. A 45-year-old man with a giant unfunctional pituitary adenoma. CC has severe visual field defects, ophthalmoplegia, hypopituitarism, and severe headache. **(A–C)**, Preoperative coronal **(A)**, sagittal **(B)**, and axial **(C)** T1-weighted contrast-enhanced MRI showing a pituitary adenoma with left-side cavernous sinus invasion (Knosp grade 4) and extension through the superior (open arrow, in **A, B**), lateral (solid arrow, in **A, C**), and posterior (solid arrowhead, in **B, C**) compartments. The disorderly extension of the tumor had caused compression in basal ganglia**(A)** by the superior aspect of the tumor. It compressed the midbrain (cerebral peduncle) by the posterior aspect **(B, C)**. It caused a significant displacement of the temporal lobe **(A, C)**. The tumor’s relationship with ICA (asterisk) was given, and tumor **(A, C)** encased the left side of the ICA (asterisk). After removing the intrasellar aspect (open arrowhead, in C) of the tumor, we deal with the rest part following an arrangement decided by the surgeon’s preference as “superior—posterior—lateral.” **(D–E)**, Postoperative T1-weighted contrast-enhanced MR images showing residual tumor of lateral aspect (solid arrow, in **D**) and total resection for the rest aspect (dotted arrow, in **E**). A planned secondary-stage transcranial surgery was performed to resect the residual tumor lateral portion. ICA, internal carotid artery; bas. A, basilar artery; bas.gang., basal ganglia; cere. ped., cerebral peduncle; tem., temporal.

**Figure 6:**
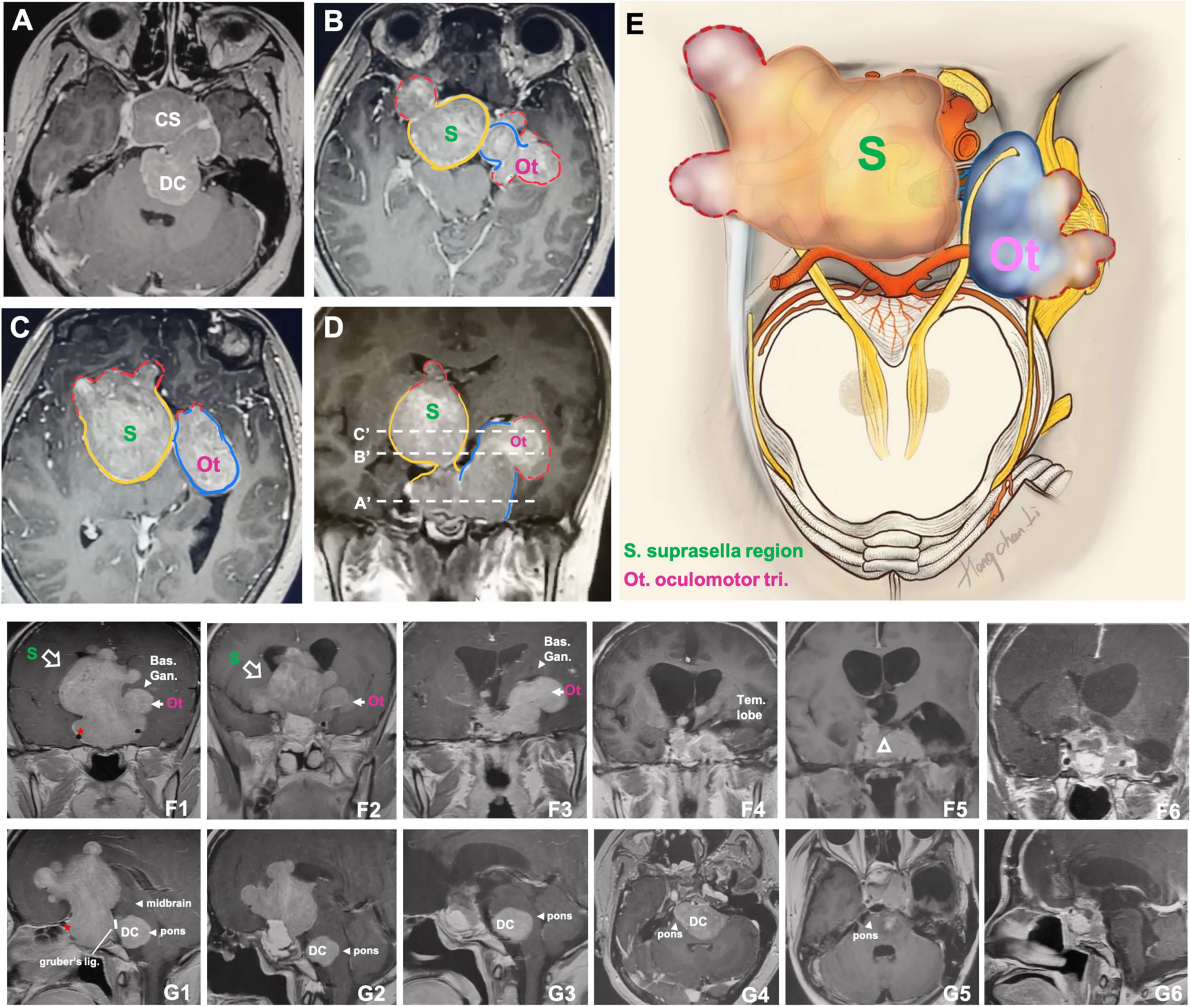
CASE5, the superior (oculomotor triangle)-posterior (Dorello’s canal) compartment extension. A 29-year-old female with a giant non-functional pituitary adenoma. CC had cessation of menstruation for five years, blurred vision for half a year with memory loss, and urinary incontinence. **(A–E)**, Preoperative axial **(A–C)** and coronal **(D)** T1-weighted contrast-enhanced MRI showing a Knosp grade 4 pituitary adenoma with complex penetration growth pattern, which can be divided into different “parts” (see diagram **E**) according to their growth path: 1) cavernous sinus invasion **(A)**; 2) superior compartment invasion: intrasellar midline growth) **(B, C)** cause suprasellar septum lifting (represented by “S”), and oculomotor triangle penetration (represented by “Ot”); 3) posterior compartment invasion (A, G1-4) cause dorello’s canal penetration (represented by “DC “) and brainstem compress. A red dot line outlined subarachnoid Penetration in **B-D**. **(D)** (A ‘, B,’ and C’ planes correspond to **A–C**, respectively). We performed Five-staged operations F, G on this case and finally achieved STR of the tumor. Pre-operation MRI(F1,G1), ICA (asterisk); 1st-time: resect intrasellar and suprasellar tumors by EEA+TC, post-op.MRI(F2, G2); 2nd-time: resect midline growth (the “S”) part by TC, post-op.MRI(F3,G3); 3rd-time: resect tumor in left temporal lobe+cavernous sinus+ petroclival region by TC(Dolenc+Kawase), post-op.MRI(F4, G4); 4th-time: resect tumors in dorello’s canal (the “DC” part) in petroclival region by TC, post-op.MRI(F5,G5); 5th-time: resect residual tumor(open triangle) by EEA, post-op.MRI(F6,G6). bas.gang., basal ganglia; tem., temporal; oculom. trai., oculomotor triangle; D.C., dorello’s canal; EEA, endonasal transsphenoidal approach; post-op., post operation; STR, sub-total resection; TC, transcranial approach.

This section explores and illustrates five different penetration patterns. A hoop-like shape that formed on the tumor body was identified as a sign of Penetration through the natural narrow entrance. Superior Penetration often manifested as directionality toward (1) midline (**Figure 3A** and cases 4, 5); (2) oculomotor triangle (**Figures 3A**, **4B** and case 5), causing displacement of the optic nerve (CN II) medially (**Figure 3G**), and oculomotor nerve (CN III) laterally (**Figure 3H**), (3) Dolenc’s triangle (**Figure 2**). With lateral penetration, medial temporal lobe compression was commonly found (case 4), but peripheral ligament integrity should be distinguished carefully to confirm the of invasion (**Figure 4C**), and the degree of Penetration may affect the choice of EEA (**Figure 3B**) or TC. In our series, grade 3 required a planned second-stage surgery for complete removal (**Figure 5D**). Two well-defined regions of posterior Penetration were demarcated by the petrosphenoidal ligament (Gruber’s ligament). When Meckel’s cave was carefully opened, and the Gruber’s ligament was cut off, the tumor extended into Dorello’s canal (**Figures 6A**, G1–4) and was reached by the TC. In contrast, the tumor penetrated the CS posterior wall and caused ventral cerebral peduncle compression (**Figure 4A**) of Gruber’s ligament dorsally.

All cases were classified into Lou’s grading system: grade 1: cases 1 and 2; grade 2: case 3; and grade 3: cases 4 and 5. Four patients (cases 1-4) remained stable at the last follow-up (ranging from 12 months to 7 years) with no tumor progression, and one case died from asphyxia due to grand mal seizures at home on the sixth month of post-operational follow-up (case 5).

## 4 Discussion

Surgery for invasive pituitary adenomas is an interdisciplinary and challenging part of the surgical technique. This work described five intracranial extension subtypes formed by superior/lateral/posterior cavernous sinus compartment penetration of Knosp4PA. Understanding the “sella turcica -cavernous sinus invade and penetration-intracranial extend” process helps analyze imaging studies with greater detail to identify the potential location of intracranial extension and therefore facilitates planning the surgical approach to each intracranial region.

Preoperative planning and decision-making about the optimal individualized strategy for invasive Knosp4PA are usually based on preoperative imaging (12–15). In the face of these tumors’ vast size and unusual, asymmetric intracranial extent, the standard surgical approaches must be modified or combined to expose and resect the tumor in the best possible fashion (16, 17). In this, all Knosp four series, 10% (11 cases) intracranial extension received staged surgery. EEA was adopted as the first surgical option, practiced in 100%, 86%, 93%, and 94% of different grades of intracranial extension and the management of extensions localized outside the sella turcica (18). These results suggest a good practice of subdivision of intracranial extensions, which benefit from tracing along the perforations it has created during its expansion (19, 20). With a reduced rate of transcranial surgery, the inherent risk of damage to the surrounding structures (16, 18), which must be exposed and dissected, such as the pituitary gland, blood vessels, and the visual pathways may also go down.

On the four lateral walls defined by Rhoton, weak ligament spaces and cranial nerve inlets as providing natural channels connecting the cavernous sinus and the middle skull base (21), from where the invasive pituitary adenomas can penetrate through the cavernous sinus and grow outward (22–24). For different walls of the cavernous sinus, the most commonly invaded part was the superior compartment, followed by the lateral and posterior compartments (68.4% *vs*. 59.2% *vs*. 28.4%) (**Table 3**). This may be related to the fact that the superior wall of the CS is formed by three triangles and is made up of one dural layer, and the lateral and the posterior wall of the CS are both consist of two dural layers (25–27). Despite different types of intracranial extension may exist randomly (4), the results showed that the oculomotor triangle was still the most easily penetrated area 24.2%(29/120 cases) compared with any other areas, such as Parkinson’s triangle(18.3%), cerebral peduncle(5.8%), Dolenc’s triangle(5%), and Dorello’s canal (1.7%).

The number of GTR PAs is also affected by the degree of tumor invasion into the CS cavity. G. Woodworth et al. (28) found a significant difference between the GTR of PAs with Knosp grade 1-2 invasion and the GTR of tumors with grade 3-4 invasion (84.6% *vs*. 66.6%, p=0.04). In addition, it seems unreasonable to evaluate all Knosp4PAs together, as P.L. Kalinin (29) noted that the number of total resections of PAs invading the CS cavity is much lower than in the absence of latero-sellar tumor extension (51% *vs*. 86.9%). In this case series, the total GTR was 50% (19-82%) (n=60), and the total NTR was 31.6% (n=38). It is worth noting that the GTR decreased as the grade increased (according to Lou’s scale) and presented a significant difference between single and multiple CS compartment involvement (36.7 *vs*. 13.3%, P<0.05) (**Table 4**) and statistical difference in GTR between grade0 and grade2 (p<0.05), grade3 (p<0.05). These may suggest that GTR is more challenging to achieve with multiple CS compartment invasions and a more significant tumor penetration grade (>2 cm, grade 2). According to our subclassification scheme, the tailored surgical strategies suggested that EEA was preferred to remove superiorly and posteriorly penetrating grade 0-3 and laterally penetrating grade 0-2 tumors. For tumors with Dolenc triangle (case 1) and Dorello canal extension (case 5), EEA combined craniotomy is recommended, as well as in lateral grade 3 extension (case 4), because of the limited vision of lateral exposure beyond the CNs and ICA (30).

The dynamics of visual function improvement (68.1%) are used as significant indicators of safety and efficacy. Particular attention was given to patients with an oculomotor triangle (24.2%) and posterior triangle penetration (24.2%) because procedures from the posterior-inferior and anterior-inferior portions of the lateral ICA can increase the risk of CNVI damage. In contrast, removing the posterior-superior and anterior-superior portions and the lateral aspect of the internal carotid artery predisposes patients to CNIII damage (8). When patients had only their ipsilateral-side vision affected, we performed only decompression surgery to ensure that the patient’s oculomotor nerve was not damaged. The incidence of new transient and permanent CN palsy was lower than in the previous data. H. Nishioka (3) reported that the probability of hormone remission of functional adenoma invading the cavernous sinus decreased step by step from Knosp1-4 (90-0%), so hormone replacement therapy with the monitoring of hormone deficiency is crucial considering the massive PA invasion in this series. ICA injury is a rare but potentially fatal complication that occurs in 0-3.8% of cases (31). We actively repaired the ICA during the operation in one patient (0.8%). EEA can provide a panoramic view of the surgical field under good lighting conditions and minimum invasion (30), so resection strategies that follow the tumor growth trajectory can be achieved, emphasizing the rationality of advanced EEA.

### 4.1 Limitations

This study has some limitations. Firstly, it was a retrospective study and so had inherent selection bias. Besides, the penetration classification needs more clinical evaluation to better understand its clinical-surgical significance. Furthermore, the influence of gamma knife adjuvant therapy, repeated surgery, and hormone replacement needs further evaluation, especially under this proposed classification.

## 5 Conclusion

This new classification based on MRI, anatomical traits, and clinical findings further supplements the existing Knosp, Goel, and Fernandez-Miranda classification. The fairly systematic summary of Penetration given here can help surgeons take advantage of the natural corridor provided by the tumor to achieve adequate exposure and targeted resection during EEA, which promotes the safety, efficiency, simplicity, and selection of surgical strategies.

## Data availability statement

The raw data supporting the conclusions of this article will be made available by the authors, without undue reservation.

## Ethics statement

The studies involving human participants were reviewed and approved by the Ethics committee of Shanghai General Hospital, affiliation to Shanghai JiaoTong University, school of medicine. The patients/participants provided their written informed consent to participate in this study.

## Author contributions

FY, YB, QYZ, and ML contributed to the conception and design of the study. YX and QWZ organized the database. HL and JY performed the statistical analysis. FY wrote the first draft of the manuscript. FY, YB, and QYZ design illustrations are included in this article. HL drew artistic illustration in case 2 and case 5. ZW’s team performed and provided all image data. ML did all surgery. All authors contributed to the article and approved the submitted version.
