# Supplementary material for: Pituitary adenoma with cavernous sinus compartment penetration and intracranial extension: surgical anatomy, approach, and outcomes
Source: Front Oncol. 2023 May 18;13:1169224. doi: 10.3389/fonc.2023.1169224 (PMC10232909; doi:10.3389/fonc.2023.1169224)
Supplement: Supplementary file 1 [file DataSheet_1.docx]

video 1 (illustrated case 2/figure3 in manuscript): showing transcranial surgery for the removal of Knosp4PA with the superior (midline + oculomotor triangle) and lateral compartments penetration.


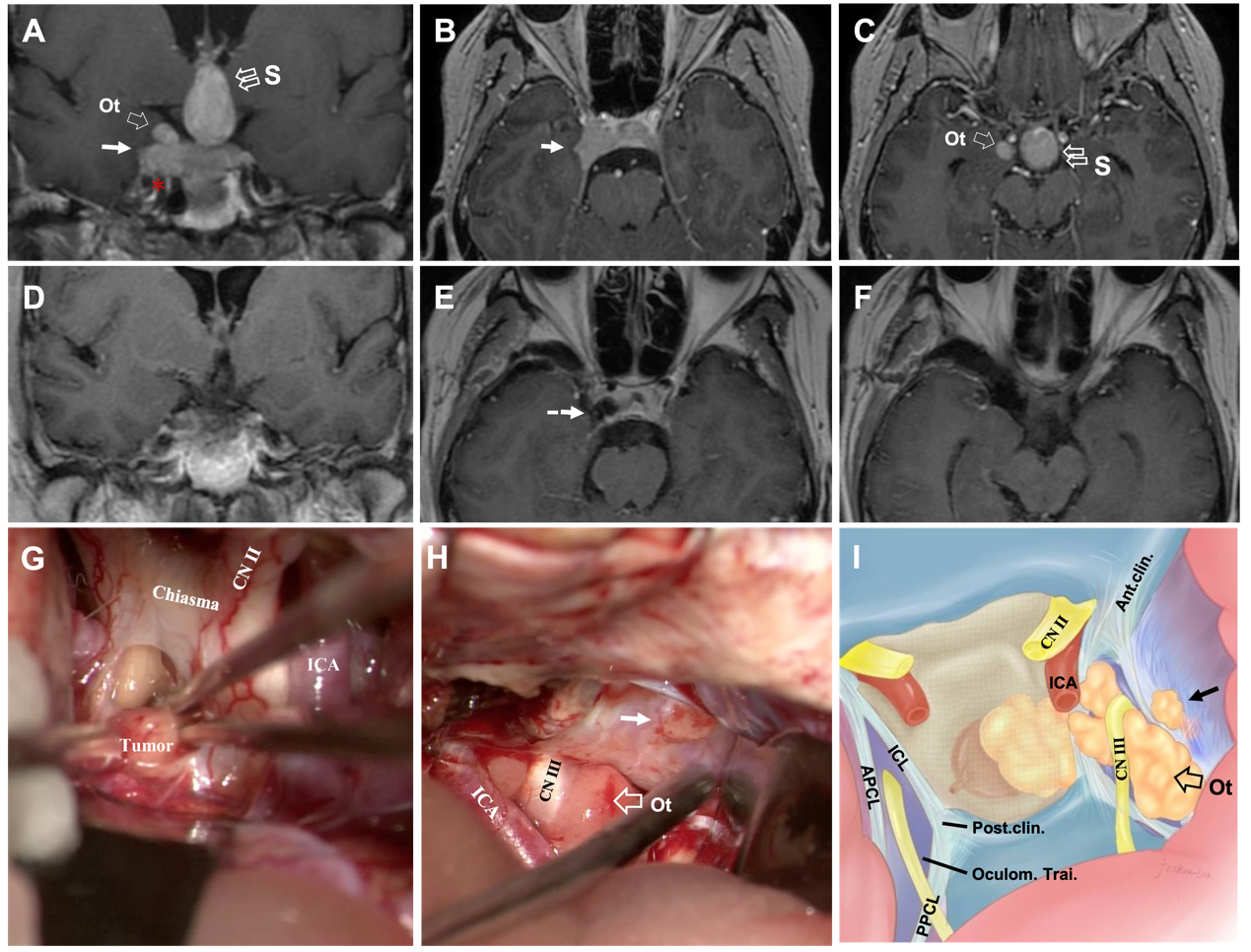


Figure3 (case 2) in manuscript, corresponding to video 1.

video 2 (illustrated case 4/figure5 in manuscript): showing EEA for the removal of Knosp4PA with the superior-posterior-lateral compartment penetration.
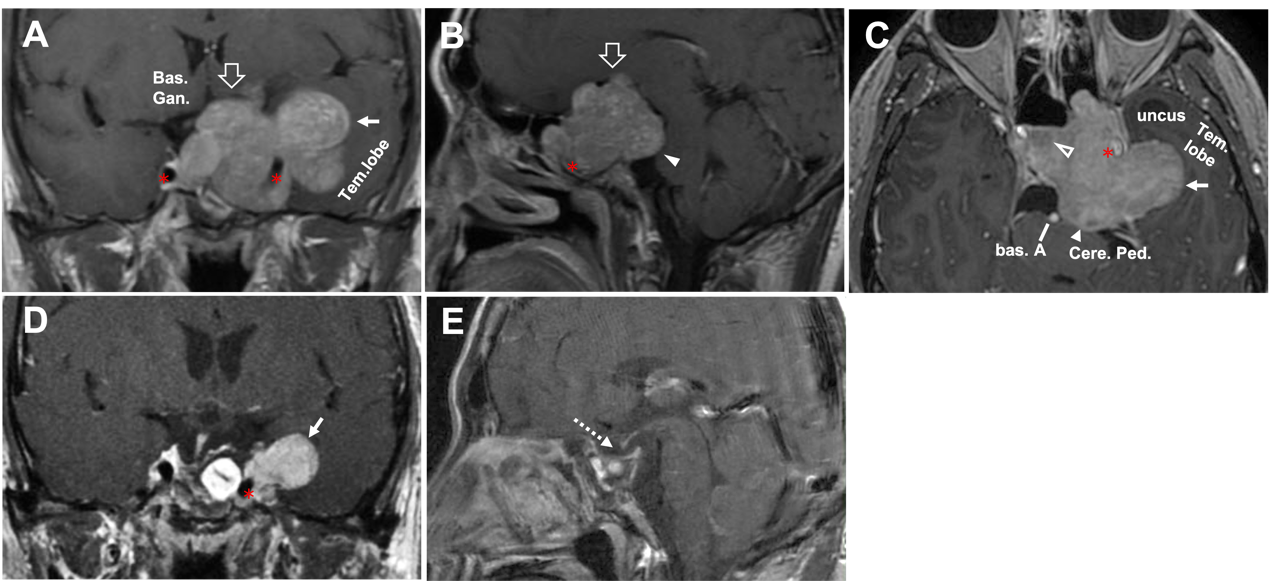


Figure5 (case 4) in manuscript, corresponding to video 2.
